# Executive Function and Postural Instability in People with Parkinson's Disease

**DOI:** 10.1155/2014/684758

**Published:** 2014-07-17

**Authors:** Dong Xu, Michael H. Cole, Kerrie Mengersen, Peter A. Silburn, Feng Qiu, Cara Graepel, Graham K. Kerr

**Affiliations:** ^1^Movement Neuroscience Program, Institute of Health & Biomedical Innovation, Queensland University of Technology, Brisbane, 60 Musk Avenue, Kelvin Grove, QLD 4059, Australia; ^2^School of Exercise and Nutrition Sciences, Queensland University of Technology, Brisbane, 60 Musk Avenue, Kelvin Grove, QLD 4059, Australia; ^3^Department of Neurology, Shandong Provincial Hospital, Jinan, Shandong 250021, China; ^4^School of Exercise Science, Australian Catholic University, Brisbane, QLD 4014, Australia; ^5^School of Mathematical Science, Queensland University of Technology, Brisbane, QLD 4059, Australia; ^6^University of Queensland Centre for Clinical Research, Brisbane, QLD 4059, Australia; ^7^Department of Neurology, Affiliated Nanjing Brain Hospital, Nanjing Medical University, Nanjing, Jiangsu 210029, China

## Abstract

The specific aspects of cognition contributing to balance and gait have not been clarified in people with Parkinson's disease (PD). Twenty PD participants and twenty age- and gender-matched healthy controls were assessed on cognition and clinical mobility tests. General cognition was assessed with the Mini Mental State Exam and Addenbrooke's Cognitive Exam. Executive function was evaluated using the Trail Making Tests (TMT-A and TMT-B) and a computerized cognitive battery which included a series of choice reaction time (CRT) tests. Clinical gait and balance measures included the Tinetti, Timed Up & Go, Berg Balance, and Functional Reach tests. PD participants performed significantly worse than the controls on the tests of cognitive and executive function, balance, and gait. PD participants took longer on Trail Making Tests, CRT-Location, and CRT-Colour (inhibition response). Furthermore, executive function, particularly longer times on CRT-Distracter and greater errors on the TMT-B, was associated with worse balance and gait performance in the PD group. Measures of general cognition were not associated with balance and gait measures in either group. For PD participants, attention and executive function were impaired. Components of executive function, particularly those involving inhibition response and distracters, were associated with poorer balance and gait performance in PD.

## 1. Introduction

Postural instability and gait disturbance are a major concern for people with PD, particularly given their relationship to an increased risk of falls in this population [1, 2]. Although clinical tests of balance and gait have been widely used to assess functional capacity and fall risk in older people [3] and people with PD [1, 4], less attention has been given to the possible contribution of cognitive function to postural instability in the PD population [5, 6].

Cognitive impairments are as common as gait and balance disturbances in people with PD, with prevalence six times greater than the elderly [7]. Across the range of cognitive domains, several studies have reported impairments in executive function for people with PD [8], specifically impaired set-shifting ability [9] and response inhibition processes [10].

Previous evidence has suggested that executive dysfunction is associated with poorer gait and balance performance [11] and increased falls risk [12] in older adults. Similarly, impaired response inhibition and slower responses to distracter stimuli are also associated with poorer balance and gait performance in older people [13]. However, to our knowledge, the way in which these specific aspects of cognition affect balance and gait in people with PD is yet to be determined.

Few studies have examined the impact of cognition on gait and balance in people with PD [14, 15]. Yogev et al. [14] observed that declines in executive function in people with PD were associated with increased gait variability while performing a concurrent cognitive task. Furthermore, a separate study has shown that practicing performing a concurrent cognitive task while walking may be effective in improving characteristics of gait in people with PD [16]. Nevertheless, the existing research presents contradictory arguments regarding the role of cognition in predicting falls risk in PD individuals [17]. Therefore, in order to design effective future interventions, there is a need to comprehensively investigate whether executive function is related to postural instability and gait disturbance in this population.

The current study evaluated balance and gait performance utilizing a combination of commonly used clinical assessments validated in PD [18–20]. These included the Tinetti balance and gait [3], Timed Up & Go (TUG) [21], Berg Balance Scale (BBS) [22], and Functional Reach (FR) tests [23]. We aimed at exploring any differences in executive function and attention aspects between people with PD and healthy adults and at examining the relationship between their cognitive profiles and clinical measures of balance and gait. It was hypothesized that people with PD would perform worse on measures of executive function and the assessments of balance and gait. Furthermore, poorer performance on measures of cognitive function would be correlated with poorer balance and gait performance.

## 2. Methods

### 2.1. Participants

Twenty participants with PD who fulfilled the UK Brain Bank criteria and were assessed as having mild-to-moderate disease severity were enrolled from community support groups and neurology clinics in southeast Queensland from June 2010 to December 2010. During this time period, twenty age- and gender-matched healthy controls were recruited from a preexisting database of people who had expressed an interest in participating in research. Participants were excluded if they had undergone deep brain stimulation surgery, were unable to walk independently, or had (i) a recent history of cardiovascular problems, (ii) injuries or surgery, (iii) any uncorrected visual disturbances, (iv) other known neurological or psychiatric disorders, or (v) significant cognitive impairment (Mini-Mental State Examination (MMSE) total score < 25 [24]). The study was approved by the Uniting Care and Queensland University of Technology Human Research Ethics Committees in Brisbane, Australia. All participants provided informed written consent to participate in the study.

### 2.2. Procedures

#### 2.2.1. Baseline Assessments

To evaluate disease state and symptom severity, PD participants were clinically evaluated using the Unified Parkinson's Disease Rating Score (UPDRS), the Hoehn and Yahr scale, and the Schwab and England Activity of Daily Living scale (S&E ADL) [25]. Additionally, the extent of any gait difficulties was evaluated using the Freezing of Gait (FOG) questionnaire [26] and the Postural Instability and Gait Disability (PIGD) score derived from the UPDRS (sum of items 13 to15 and 27 to 30). PD participants were classified into tremor dominant, akinetic-rigid, or mixed subtypes based on UPDRS items according to the method described by Kang et al. [27]. Tremor scores were derived from the sum of UPDRS items 20 and 21 divided by 4. Rigidity scores were derived from the sum of items 22 to 27 and 31 divided by 15. Based on the ratio of tremor scores to rigidity scores, 10 PD participants were classified as being of the akinetic rigid type, 7 PD participants were tremor dominant, and 3 were considered to be of the mixed subtype [27].

Addenbrooke's Cognitive Examination (ACE) [28], which incorporates the MMSE score, was administered to all participants prior to testing to identify any participants with dementia. This general cognitive test evaluates five cognitive domains including attention/orientation (date and place orientation, number subtraction, and spelling words backwards), memory (anterograde and retrograde memory and recall), fluency (verbal fluency and category fluency, e.g., types of animals), language (comprehension, naming, reading, writing, and repetition), and visuospatial skills (clock drawing and copying a diagram, e.g., pentagons and perceptual ability). PD participants performed all assessments while optimally medicated.

During a second visit, seventeen participants (10 PD and 7 controls) derived from the above cohort also completed the computerized cognitive battery described previously [13, 29].

#### 2.2.2. Clinical Balance and Gait Assessments

To clinically evaluate balance and gait, the participants were assessed using the Tinettitest, which comprises a balance (TBS maximum score = 16) and gait score (TGS maximum score = 12), which are combined to provide the Tinetti total score (TTS maximum score = 28). Similarly, the BBS was used to assess balance while performing 14 tasks that are common of daily living (maximum score = 56) and the FRtest was used to measure the maximum distance that the participants could reach without losing their balance in a standing position. To assess mobility, participants completed the TUG test, which measures the time taken for the participant to stand from a seated position, to walk at a comfortable pace to a distance 3 m away, to turn around, and to return to the chair to sit down.Higher scores on the TBS, TGS, TTS, BBS, and FR indicate better performance, while shorter times for the TUG indicate better performance.

#### 2.2.3. Executive Function and Attention Measures

*(1) Trail Making Test (TMT).* Participants were required to connect consecutive circled numbers for the TMT-A and to connect numbers and letters in an alternating sequence for the TMT-B. In both tasks, the time taken and the number of errors were recorded. The difference between the time taken to complete the TMT-A and TMT-B (TMT B-A) was calculated to represent executive function controlling for processing speed. Greater time and errors on the TMT indicated poorer executive function [30].

*(2) Computerized Cognitive Battery.* The computerized battery included a series of reaction time (RT) tests, the Digit Symbol Match test, and the Self-Ordered Pointing Test which has previously been used to assess older adults [13]. To familiarise participants, a series of practice trials were performed for all tests before commencing.

*Reaction Time Tests.* Participants were instructed to perform the tasks using a response box (3 buttons) and two pedals. Two buttons on the response box and the two pedals were used in all CRT tasks, and the additional middle button on the response box was used in the CRT tasks that included distracters [13].

For the* Simple RT *(SRT) tests,a stimulus (image of a car) was presented 30 times at random intervals on the screen. Participants were required to respond by pressing the button as quickly as possible with their dominant hand.* The Choice RT *(CRT) test used the same visual stimulus, which was randomly presented during 40 trials in one of the four corners of the screen. Participants responded by pressing the button or pedal corresponding to the quadrant that the car was displayed in. The* Choice RT Location *(CRT-L) and* Choice RT Colour *(CRT-C) tasks were similar to the CRT task but, for these tests, participants were asked not to respond if the target stimulus appeared either in a specific location (in the top right corner) or as a specific colour (a blue car). There were 40 trials for the CRT-L and 64 trials for the CRT-C, which, respectively, included 10 and 16 “no response” trials (inhibition responses). The* Choice RT Distracter* (CRT-D) test was similar to the other CRT tasks, but a “stop” sign randomly appeared in the centre of the screen following a presented stimulus. If the “stop” sign appeared on the screen, participants were required to press the additional middle button on the response box after pressing the corresponding button or pedal. Average RTs for the correct responses (CRT-DT) and for the time taken to respond to the stop sign (CRT-DS) were recorded. The CRT-D test included 60 trials, 16 of which involved presentation of the stop sign (distracter).

For all of the RT tasks, only correct trials were included for calculation of average RTs and any values that lay further than ±3 SD from the mean were replaced with a value equal to the mean ±3 SD [29]. The numbers of response inhibition errors were also recorded for the CRT-L and CRT-C tasks.

*Digit Symbol Match (DSM).* A list of matching pairs of symbols and numbers was presented at the top of the screen. Participants were required to press “yes” if the pairs matched or “no” if the pairs were unmatched. There were 72 pairs of symbols presented in the task and the mean RT for correct responses and the accuracy of the responses were measured.

*Self-Ordered Pointing Test (SOPT).* Participants were presented with 12 different patterns. Participants were instructed to select a pattern on the first presented display, after which the display was shuffled and the participants were asked to select a different pattern on the next display. This process was repeated until all 12 original patterns had been selected without selecting the same pattern twice. The number of errors (selecting a pattern more than once) was measured.

### 2.3. Statistical Analysis

Descriptive statistics were calculated for each of the continuous variables derived from the assessments of cognition, executive function, balance, and gait, and the normality of the data was determined. Independent two-tailed* t*-tests were used to examine mean difference between the two groups for the assessments of cognition, balance, and gait. The Mann-Whitney *U* test was used to examine the difference between the two groups for the tests of cognition, balance, and gait that were not normally distributed. The Chi-square test was used to compare the two groups on categorical variables. Correlations between cognition, balance, and gait measures and UPDRS derived scores were tested with Spearman's correlation coefficient. All statistical analyses were performed using the Statistical Package for Social Sciences (SPSS 17.0 for Windows) and the significance level was *P* < .05.

## 3. Results

The age and years of education were similar for the two groups and PD participants were predominantly in the early stages of the disease (Table 1).

### 3.1. Balance and Gait Performance

Compared with healthy controls, PD participants performed significantly worse on all measures of balance and gait (Table 1).

There were significant correlations between the UPDRS motor score and all of the balance and gait tests except FR. There were also significant correlations between the PIGD score and all of the balance and gait tests (*P* < .001) except FR (Table 2).

### 3.2. Cognitive Function Measures

#### 3.2.1. General Cognitive Measures

Compared with controls, PD participants had a significantly lower mean MMSE and ACE total score and lower ACE attention and orientation subscores (Table 3).

#### 3.2.2. Trail Making Tests

PD participants took significantly longer on the TMT-A, TMT-B, and TMT B-A than controls. On the TMT-A, only one PD participant made a single error, while control participants recorded no errors. For the TMT-B, thirteen PD participants made at least one error and six made greater than two errors, whereas only three control participants made a single error (*P* = .003) (Table 3).

#### 3.2.3. Computerized Cognitive Battery

Compared to controls, the PD participants had longer RTs for the response inhibition tasks during the CRT-L (*P* = .028) and CRT-C (*P* = .023) tests (Table 4). Neither group made errors during the CRT-C task, but one PD participant made an error during the CRT-L test and three PD participants made errors during the CRT-D task.

### 3.3. Correlations between Executive Function and Balance and Gait

For all correlations, PD and control groups were examined separately due to the observed differences. For the PD participants, longer times for the TMT-A, TMT-B, and TMT B-A and a greater number of errors on the TMT-B were all significantly associated with poorer performance on the TBS. TMT-B errors were correlated with poorer performance on the TGS (*r* = −.513, *P* = .021), while longer times on the TMT-B and TMT B-A were associated with poorer performance on the BBS (*r* = −.464, *P* = .039 and *r* = −.523, *P* = .018). Furthermore, the time to complete the TMT-A was positively associated with performance on the TUG test in the PD group (*r* = .509, *P* = .026).

For the PD participants, slower RTs for the CRT-DT and CRT-DS tests were significantly correlated with lower scores on the BBS (*r* = −.717, *P* = .020 and *r* = −.779, *P* = .008) and TGS tests (*r* = −.747, *P* = .013 and *r* = −.735, *P* = .016) and slower performances on the TUG test (*r* = .806, *P* = .005 and *r* = .661, *P* = .038). Additionally, the CRT-L times were negatively associated with the TBS scores (*r* = −.750, *P* = .012) and the DSM measure was positively correlated with the TUG test (*r* = .733, *P* = .016) (see Supplementary Table 7 in Supplementary Material available online at http://dx.doi.org/10.1155/2014/684758).

There was no correlation between any of the executive function measures and tremor scores. However, there were significant correlations between executive function measures and the rigidity scores, PIGD scores, and UPDRS motor scores (see Table 5). Similarly, longer time on CRTs, particularly CRT-L and CRT-DT, was associated with higher PIGD scores, UPDRS motor scores, and rigidity scores whereas there was no correlation between CRTs and tremor score (see Supplementary Table 7).

There were no significant correlations between any of the cognitive measures and balance and gait performance in the control group. Similarly, there were no significant correlations between the measures of general cognition (ACE, MMSE) and balance and gait for either group (Tables 5 and 6).

## 4. Discussion

This study investigated the association between cognition, particularly executive function and attention and balance and gait for people with PD and healthy older adults. The results showed that cognition, executive function, and balance and gait were all impaired in people with PD. Furthermore, the observed impairments in executive function and attention were associated with poorer performance on the balance and gait assessments for this population.

Our results revealed that global cognition (MMSE and ACE total scores) was significantly reduced for PD participants compared to controls. The mean MMSE score for the PD group was 27.6, which was comparable with the results (27.5) presented in a previous study [32], but different from another study in which similar MMSE scores (28.5) were reported for PD participants and controls [15]. Although a few studies have identified cognitive impairment as an independent predictor of falls risk in people with PD [33], global cognitive function (MMSE scores) has not previously been reported to be significantly different between PD fallers and nonfallers [1]. In accordance with this, the present study found that the measures of global cognitive deficit (MMSE and ACE total scores) were not associated with poorer balance and gait performance in this population.

With respect to the different cognitive domains, PD participants took significantly longer to complete the TMT-A, which indicates deficits in attention and processing speed. This finding was supported by the attention/orientation subscore of the ACE, which was also significantly reduced for the PD participants. These findings agree with previous research that has shown that the TMT-A and attention index are both decreased in people with PD [15].

Executive function could be summarized into three factors that include set-shifting, inhibition, and updating [34], and the TMT-B primarily evaluates set-shifting ability. We observed that PD participants took significantly longer on the TMT-B and TMT B-A compared to controls. These results were partially consistent with a previous study [15], which reported that TMT-B times were significantly longer for PD participants. In contrast, this study reported that TMT B-A times were not significantly different to normative data; this disparity may be a result of the authors constraining the sample to people with PD who presented with motor response fluctuations [15]. Importantly, our results showed that PD participants recorded more errors on the TMT-B, suggesting that this could also be a sensitive indicator for identifying deficit of executive function in people with PD.

Inhibition response may be an important component of executive function, as it would allow people to focus on maintaining balance during walking by ignoring concurrent distractions from the environments [35]. In two of the CRT tests (CRT-C and CRT-L) that involved inhibiting responses, the PD participants recorded longer times than controls, suggesting impaired response inhibition in PD participants. This finding is consistent with a study that used the same battery to assess older adults and observed that fallers performed worse than nonfallers on tests of CRT-C and CRT-L [13].

This study demonstrated that executive function rather than general cognition was correlated with poorer balance and gait performance for people with PD. Similarly, Plotnik et al. [15] reported that executive abilities were correlated with gait performance in PD individuals. We also observed that the TMT B-A measure was significantly associated with balance scores and that PD participants who made more errors on the TMT-B were likely to perform poorer on the TGS. This finding indicated that errors on the TMT-B could provide insight into executive function deficits as it excludes the influence of processing speed. Furthermore, the findings suggest that executive function may play a more important role in the balance and gait of people with PD than general cognition.

Importantly, PD participants with longer RTs on the CRT-L test were likely to have poorer balance according to the TBS. Furthermore, slower RTs on the CRT-DT and CRT-DS (presence of distracter) were correlated with poorer performance on the TGS, TTS, and BBS and with longer times on the TUG. These findings indicated that the impaired response inhibition evident for the PD participants may impact their capacity to walk effectively, particularly in environments with many distracters.

There were distinct differences in which PD subtypes were associated with decreased executive function. Increased tremor severity was associated with an increase in overall cognitive decline (high tremor score was associated with a low MMSE/ACE score) but not with decreases in executive function. However, increases in both akinetic rigidity and PIGD were both associated with decreased executive function. Interestingly, the PIGD score was correlated with the TMT (B-A) scores which is the best indicator of executive function deficit [15]. The correlations of UPDRS (III) motor and rigidity scores were mainly related to the TMT time for the A and B tests, which is indicative of overall movement slowing rather than executive function deficit. In fact, the pattern of rigidity correlations was very similar to those of the PIGD correlations but this was most likely because they share several UPDRS variables in common so they are not entirely independent. Increased rigidity was also more related to slower RT measures associated with executive tasks. However, general movement slowing, as would be indicated by increased rigidity, may also partly explain the correlation (as per significant SRT and CRT correlations). Because the PIGD calculation also utilises items from the UPDRS (III) that are reflective of posture and gait, similar correlations with the clinical balance measures would be expected.

One of the strengths of this study was that it assessed participants using a combination of commonly used clinical balance and gait tests, while previous studies have typically only employed one of these tests (e.g., TUG [15]). It is noteworthy that PD participants performed worse on all clinical balance and gait assessments in the current study. Interestingly, performance on the TUG test was more affected by deficits in processing speed (longer TMT-A and DSM times) and less by executive capacities (e.g., TMT-B errors or TMT B-A) for the PD group. This suggests that hand mobility (TMT-A) and body movements (TUG) are correlated, which is supported by recent research showing that inhibition evaluated using the Stroop test was not related to mobility (TUG) in this population [5]. The lack of any significant correlations between cognition and the FR test may be due to the fact that FR is not known to be a sensitive predictor of falls risk in people with PD [36], while the Tinetti and BBS tests are known to be predictive of falls in this population [1].

We acknowledge that this study has a number of limitations and these should be considered in light of the results. Firstly, the relatively small sample size may have affected our capacity to detect some relationships between cognitive function and balance and gait. Secondly, although the clinical balance and gait evaluations are validated, reliable, and widely used, these scales involve somewhat subjective factors. Nevertheless, this preliminary study provides a promising direction for future by improving our understanding of the relationship between balance and gait disturbances and executive dysfunction in PD, which may have significant implications for the improved quality of life of these people.

## 5. Conclusions

Attention and executive function were impaired in people with PD, and particularly the components of executive function involving set-shifting and inhibition response, compared with healthy controls. TMT-B could be a sensitive indicator for identifying deficit of executive function in people with PD. Furthermore, the impairments in executive function and attention were associated with poorer performance on the balance and gait in PD patients. The results suggested that executive function may play a more important role in the balance and gait of people with PD than general cognition.

## Supplementary Material

The Supplementary Materials portray the correlations between the computer-based assessments of executive function and attention and the clinical measures of balance and gait performance for the PD participants and the controls. The data presented for the PD participants in Table 7 also includes the correlations between these computerized measures of cognition and subscores derived from the Unified Parkinson's Disease Rating Scale (UPDRS).

## Figures and Tables

**Table 1:** Demographic characteristics of PD and control groups.

	PD (*n* = 20)	Controls (*n* = 20)	*P* value
Demographics			
Age (yrs)	65.9 (9.4)	68.9 (4.8)	.209
Male (%)	65	65	
Education (yrs)	12.4 (2.5)	12.7 (3.4)	.752
PD participants characteristics			
Disease duration (yrs)	6.0 (3.8)		
UPDRS I	2.8 (2.2)		
UPDRS II	11.4 (7.4)		
UPDRS III	26.6 (10.8)		
UPDRS total	40.8 (17.8)		
H-Y stage	1.4 (0.9)		
FOG scores	2.0 (0–14.0)^∧^		
PIGD score	4.5 (3.6)		
Tremor score	1.0 (0.7)		
Akinetic/rigidity scores	1.2 (0.5)		
ADL (%)	86 (8.4)		
PD medications			
Levodopa (numbers taken)	14		
Dopaminergic agonists (numbers taken)	8		
COMT inhibitor (numbers taken)	4		
Monoamine oxidase inhibitor (numbers taken)	1		
Benzodiazepine (numbers taken)	2		
Serotonin reuptake inhibitors (numbers taken)	1		
No medication (numbers)	4		
LED (mg)	622.25 (477.17)		
Balance and gait performance			
TBS score	15 (8–16)	16 (15-16)	**<**.001^∧^
TGS score	9.9 (2.1)	11.2 (1.0)	**.016**
TTS score	23.7 (4.4)	27.2 (1.0)	**.002**
BBS score	53 (34–56)	56 (48–56)	.003^∧^
TUG (s)	10.8 (2.5)	9.30 (1.1)	**.027**
FR (cm)	24.5 (5.9)	35.7 (6.0)	**<.001**

Note: UPDRS: unified Parkinson's disease rating score, H-Y: Hoehn and Yahr scale, FOG: freezing of gait, PIGD: postural instability and gait disturbance, ADL: the Schwab and England Activity of Daily Living scale, LED: levodopa-equivalent dosage [31], COMT: catechol-O-methyltransferase, TBS = Tinetti's balance score, TGS: Tinetti's gait score, TTS: Tinetti's total score, TUG: Time Up & Go, BBS: Berg Balance Scale, and FR: functional reach. ^∧^Data are nonnormally distributed and values reported are median (range). All other data are normally distributed and values reported are mean (SD) and *t*-test. Significant *P* values are marked in bold.

**Table 2:** Correlations between UPDRS motor score, PIGD, and balance and gait tests.

	TBS	TGS	TTS	TUG	BBS	FR
UPDRS (III)	**−.804 (<.001)**	**−.635 (.003)**	**−.731 (<.001)**	**.701 (.001)**	**−.657 (.002)**	−.229 (.332)
PIGD	**−.771 (<.001)**	**−.809 (<.001)**	**−.841 (<.001)**	**.725 (<.001)**	**−.848 (<.001)**	−.309 (.185)

Note: TBS: Tinetti's balance score, TGS: Tinetti's gait score, TTS: Tinetti's total score, TUG: Time Up & Go, BBS: Berg Balance Scale, FR: Functional Reach, and PIGD: postural instability and gait disturbance. Significant *P* values are marked in bold.

**Table 3:** General cognitive and executive function for the PD and control groups.

	PD (*n* = 20)	Controls (*n* = 20)	*P* value
General cognitive scores			
MMSE	27.6 (1.6)	28.7 (1.1)	**.016**
ACE	90.2 (6.4)	93.8 (3.8)	**.039**
Subscores			
Attention/orientation	17.1 (0.9)	17.7 (0.5)	**.013**
Memory	22.4 (3.5)	23.8 (1.7)	.107
Fluency	11.0 (2.2)	11.6 (2.1)	.428
Language	24.6 (1.0)	25.2 (1.1)	.112
Visuospatial	15.5 (11.0–16.0)	16.0 (14.0–16.0)	.340^∧^
Executive function assessments			
TMT-A (s)	48.7 (34.0–144.4)	35.4 (25.7–62.0)	.004^∧^
TMT-A (participants with errors)	1 (5%)	0 (0%)	—
TMT-B (s)	118.3 (66.4–449.2)	85.2 (43.6–250.9)	.002^∧^
TMT-B (participants with errors)	13 (65%)	3 (15%)	.003^#^
TMT B-A (s)	78.0 (16.3–304.8)	49.8 (7.0–195.7)	.015^∧^

Note: MMSE: Mini-Mental State Examination; ACE: Addenbrooke's Cognitive Examination; TMT-A = Trail making test A; TMT-B: Trail making test B; 1 PD participant made a single error and no control participants made an error on TMT-A. ^∧^Data are nonnormally distributed and values reported are median (range) and Mann-Whitney test. All other data are normally distributed and values reported are mean (SD) and *t*-test. ^#^Chi-square test. Significant *P* values are marked in bold.

**Table 4:** Computerized cognitive measures for the PD and control groups.

	PD (*n* = 10)	Control (*n* = 7)	*P* value
SRT (s)	0.287 (0.033)	0.283 (0.031)	.816
CRT (s)	0.743 (0.150)	0.674 (0.083)	.293
CRT-C (s)	0.882 (0.167)	0.731 (0.063)	**.023**
CRT-L (s)	0.850 (0.178)	0.697 (0.062)	**.028**
CRT-DT (s)	0.877 (0.198)	0.774 (0.128)	.254
CRT-DS (s)	2.109 (0.403)	1.912 (0.127)	.232
DSM (s)	2.362 (0.450)	2.213 (0.229)	.435
SOPT (scores)	2.400 (0.843)	2.000 (0.577)	.295

Note: SRT: Simple Reaction Time; CRT: Choice Reaction Time; CRT-L: Choice Reaction Time Location; CRT-C: Choice Reaction Time Colour; CRT-DT: Choice Reaction Time Distracter; CRT-DS: Choice Reaction Time Distracter with stop signs; DSM: Digit-Symbol Match; SOPT: Self-Ordered Pointing Test. All data are normally distributed and values reported are mean (SD) and *t*-test. Significant *P* values are marked in bold.

**Table 5:** Correlations between cognitive function, the gait and balance measures, and UPDRS derived scores for the PD group.

	TBS score	TGS score	TTS score	BBS score	TUG (s)	FR (cm)	UPDRS (III)	Tremor score	Rigidity score	PIGD
ACE score	.371 (.107)	.241 (.306)	.283 (.227)	.337 (.146)	−.370 (.119)	−.073 (.760)	−.364 (.115)	**−.509 (.022)**	−.187 (.429)	−.290 (.215)
MMSE score	.310 (.184)	.290 (.215)	.288 (.217)	.143 (.546)	−.366 (.123)	−.078 (.744)	**−.453 (.045)**	**−.571 (.009)**	−.336 (.147)	−.149 (.531)
TMT-A (s)	**−.609 (.004)**	−.370 (.109)	**−.491 (.028)**	−.437 (.054)	**.509 (.026)**	−.286 (.221)	**.637 (.003)**	.266 (.257)	**.619 (.004)**	**.466 (.038)**
TMT-B (s)	**−.530 (.016)**	−.390 (.089)	**−.461 (.041)**	**−.464 (.039)**	.440 (.059)	−.281 (.230)	**.513 (.021)**	.112 (.639)	**.445 (.049)**	**.491 (.028)**
TMT-B (error)	**−.533 (.015)**	**−.513 (.021)**	**−.587 (.007)**	−.431 (.058)	.161 (.511)	−.186 (.433)	**.467 (.038)**	−.232 (.325)	**.488 (.029)**	**.478 (.033)**
TMT-B-A (s)	**−.460 (.041)**	−.412 (.071)	−.435 (.055)	**−**.**523 (.018)**	.451 (.053)	−.217 (.358)	.418 (.067)	.084 (.724)	.343 (.138)	**.486 (.030)**

Note: 1 PD participant made a single error on the TMT-A. The numbers in parentheses are *P* values. Significant correlations are marked in bold.

**Table 6:** Correlations between cognitive function and the gait and balance measures for the control group.

	TBS score	TGS score	TTS score	BBS score	TUG (s)	FR (cm)
ACE score	.217 (.373)	−.024 (.924)	.063 (.797)	.169 (.489)	.143 (559)	−.071 (.772)
MMSE score	.202 (.407)	−.305 (.204)	−.231 (.341)	−.101 (.681)	.152 (.535)	−.302 (.209)
TMT-A (s)	.129 (.598)	.337 (.158)	.398 (.091)	−.018 (.941)	.449 (.054)	−.383 (.106)
TMT-B (s)	.172 (.481)	−.021 (.931)	.046 (.853)	.053 (.829)	.356 (.135)	−.134 (.585)
TMT-B error	.102 (.678)	.201 (.410)	.243 (.317)	.077 (.755)	.395 (.094)	−.119 (.628)
TMT B-A (s)	.172 (.481)	−.214 (.379)	−.153 (.532)	.177 (.468)	.328 (.170)	−.159 (.515)

*Note*. No control participants made an error on the TMT-A. The numbers in parentheses are *P* values.
