# Supplementary material for: Executive Function and Postural Instability in People with Parkinson's Disease
Source: Parkinsons Dis. 2014 Jul 17;2014:684758. doi: 10.1155/2014/684758 (PMC4127201; doi:10.1155/2014/684758)
Supplement: Supplementary file 1 — The Supplementary Materials portray the correlations between the computer-based assessments of executive function and attention and the clinical measures of balance and gait performance for the PD participants and the controls. The data presented for the PD participants in Table 7 also includes the correlations between these computerized measures of cognition and subscores derived from the Unified Parkinson's Disease Rating Scale (UPDRS). [file 684758.f1.docx]

**Supplementary Tables**

| **Table 4:** Correlations between cognition and the gait and balance measures for the PD group | | | | | | |
| --- | --- | --- | --- | --- | --- | --- |
|  | TBS score | TGS score | TTS score | BBS score | TUG (s) | FR (cm) |
| ACE score | .371 (.107) | .241(.306) | .283 (.227) | .337 (.146) | -.370 (.119) | -.073 (.760) |
| MMSE score | .310 (.184) | .290 (.215) | .288 (.217) | .143 (.546) | -.366 (.123) | -.078(.744) |
| TMT-A (s) | **-.609 (.004)** | -.370 (.109) | **-.491 (.028)** | -.437 (.054) | **.509 (.026)** | -.286 (.221) |
| TMT-B (s) | **-.530 (.016)** | -.390 (.089) | **-.461 (.041)** | **-.464 (.039)** | .440 (.059) | -.281 (.230) |
| TMT-B (errors) | **-.533 (.015)** | **-.513(.021)** | **-.587 (.007)** | -.431 (.058) | .161 (.511) | -.186 (.433) |
| TMT B-A (s) | **-.460 (.041)** | -.412 (.071) | -.435 (.055) | **-.523 (.018)** | .451 (.053) | -.217 (.358) |

Note. 1 PD participant made a single error on the TMT-A. The numbers in parentheses are *p* values. Significant correlations marked in bold.

| **Table 5:** Correlations between cognition and the gait and balance measures for the control group | | | | | | |
| --- | --- | --- | --- | --- | --- | --- |
|  | TBS score | TGS score | TTS score | BBS score | TUG (s) | FR (cm) |
| ACE score | .217 (.373) | -.024 (.924) | .063 (.797) | .169 (.489) | .143 (559) | -.071 (.772) |
| MMSE score | .202 (.407) | -.305 (.204) | -.231 (.341) | -.101 (.681) | .152 (.535) | -.302 (.209) |
| TMT-A (s) | .129 (.598) | .337 (.158) | .398 (.091) | -.018 (.941) | .449 (.054) | -.383 (.106) |
| TMT-B (s) | .172 (.481) | -.021 (.931) | .046 (.853) | .053 (.829) | .356 (.135) | -.134 (.585) |
| TMT-B error | .102 (.678) | .201 (.410) | .243 (.317) | .077 (.755) | .395 (.094) | -.119 (.628) |
| TMT B-A (s) | .172 (.481) | -.214 (.379) | -.153 (.532) | .177 (.468) | .328 (.170) | -.159 (.515) |

Note. No control participants made an error on the TMT-A. The numbers in parentheses are *p* values.

**Table 6:** Correlations between computerized cognitive measures and the balance and gait measures for the PD group

|  | TBS score | TGS score | TTS score | BBS score | TUG (s) | FR (cm) |
| --- | --- | --- | --- | --- | --- | --- |
| SRT (s) | -.543 (.105) | -.333 (.347) | -.421 (.226) | -.312 (.381) | .418 (.229) | .03 (.933) |
| CRT (s) | -.517 (.126) | -.562 (.091) | -.591 (.072) | -.480 (.160) | .394 (.260) | .018 (.960) |
| CRT-C (s) | -.569 (.086) | -.432 (.212) | -.555 (.096) | -.287 (.422) | .455 (.187) | .396 (.257) |
| CRT-L (s) | **-.750 (.012)** | .562 (.091) | **-.689 (.028)** | -.474 (.167) | .539 (.108) | .220 (.542) |
| CRT-DT (s) | **-.763 (.010)** | **-.747 (.013)** | **-.762 (.010)** | **-.717 (.020)** | **.806 (.005)** | .189 (.601) |
| CRT-DS (s) | -.530 (.115) | **-.735 (.016)** | **-.671 (.034)** | **-.779 (.008)** | **.661 (.038)** | .073 (.841) |
| DSM (s) | -.550 (.100) | -.438 (.205) | -.463 (.177) | -.355 (.314) | **.733 (.016)** | .506 (.136) |
| SOPT(scores) | -.238 (.508) | -.447 (.195) | -.402 (.249) | -.162 (.655) | -.085 (.815) | -.475 (.165) |

Note. The numbers in parentheses are *p* values. Significant correlations marked in bold.

**Table 7:** Correlations between computerized cognitive measures and the balance and gait measures for the control group

|  | TGS score | TTS score | BBS score | TUG (s) | FR (cm) |
| --- | --- | --- | --- | --- | --- |
| SRT (s) | -.028 (.952) | -.028 (.952) | .348 (.444) | -.234 (.613) | -.054 (.908) |
| CRT (s) | -.580 (.172) | -.580 (.172) | .394 (.382) | -.750 (.052) | .143 (.760) |
| CRT-C (s) | -.661 (.106) | -.661 (.106) | .179 (.701) | -.523 (.229) | .432 (.333) |
| CRT-L (s) | -.580 (.172) | -.580 (.172) | .158 (.736) | -.286 (.535) | .429 (.337) |
| CRT-DT (s) | -.265 (.612) | -.265 (.612) | .273 (.60) | .371 (.468) | .486 (.329) |
| CRT-DS (s) | -.206 (.658) | -.206 (.658) | -.02 (.967) | .000 (1.0) | .143 (.760) |
| DSM (s) | -.617 (.140) | -.617 (.140) | -.059 (.90) | -.607 (.148) | .071 (.879) |
| SOPT (scores) | -.420 (.348) | -.420 (.348) | -.516 (.236) | -.401 (.373) | -.134 (.775) |

Note. Controls’ TBS scores were constant. The numbers in parentheses are *p* values.
